# Metabolism, Genomics, and DNA Repair in the Mouse Aging Liver

**DOI:** 10.1155/2011/859415

**Published:** 2011-04-06

**Authors:** Michel Lebel, Nadja C. de Souza-Pinto, Vilhelm A. Bohr

**Affiliations:** ^1^Centre de Recherche en Cancérologie de l'Université Laval, Hôpital Hôtel-Dieu de Québec, 9 McMahon Street Quebec City, QC, Canada G1R 2J6; ^2^Departmento de Bioquímica, Instituto de Química, Universiade de São Paulo, Avenue Prof. Lineu Prestes, 748, 05508-900 São Paulo, SP, Brazil; ^3^Laboratory of Molecular Gerontology, National Institute on Aging, National Institutes of Health, 5600 Nathan Shock Drive, Baltimore, MD 21224, USA

## Abstract

The liver plays a pivotal role in the metabolism of nutrients, drugs, hormones, and metabolic waste products, thereby maintaining body homeostasis. The liver undergoes substantial changes in structure and function within old age. Such changes are associated with significant impairment of many hepatic metabolic and detoxification activities, with implications for systemic aging and age-related disease. It has become clear, using rodent models as biological tools, that genetic instability in the form of gross DNA rearrangements or point mutations accumulate in the liver with age. DNA lesions, such as oxidized bases or persistent breaks, increase with age and correlate well with the presence of senescent hepatocytes. The level of DNA damage and/or mutation can be affected by changes in carcinogen activation, decreased ability to repair DNA, or a combination of these factors. This paper covers some of the DNA repair pathways affecting liver homeostasis with age using rodents as model systems.

## 1. Introduction

The liver plays a pivotal role in the metabolism of nutrients, drugs, hormones, and metabolic waste products, thereby maintaining body homeostasis. The liver is central to glucose and lipid homeostasis as well as steroid biosynthesis and degradation. This organ also has a major impact on health and homeostasis through its control of serum protein composition. The liver undergoes substantial changes in structure and function in old age. For example, serum and biliary cholesterol rise, liver regeneration declines, hepatic drug clearance decreases, and liver volume and blood flow decrease with advancing age [1]. Such changes are associated with significant impairment of many hepatic metabolic and detoxification activities with implications for systemic aging and age-related disease. In addition to the altered hepatocyte functions with age, major morphological changes also occur with the liver sinusoidal endothelial cells. These specialized endothelial cells are very thin and contain pores approximately 50–150 nm in diameter grouped together in clusters. This fenestration of the endothelial lining allows a wide range of substrates including larger molecules like triglyceride-rich spherical lipoproteins called chylomicrons to reach the underlying hepatocytes for processing [1]. The size and number of pores decrease with age in several mammalian species. Such changes are believed to impact directly on the hepatic metabolism of lipoproteins thus predisposing aged individuals to cardiovascular diseases [2]. Finally, all these morphological and functional changes in the liver tissue are likely to affect therapeutic interventions due to the handling and metabolism of pharmacological agents in older individuals. Adverse drug reactions are estimated to be two to three times higher in elderly patients than in young adults [3, 4].

Concomitant with morphological changes, the liver exhibits important alterations in global gene expression profiles with age. In mice, aging is accompanied by changes in expression of genes associated with increased inflammation, cellular stress, fibrosis, altered capacity for apoptosis, xenobiotic metabolism, normal cell-cycle control, and DNA replication [5]. Lifelong calorie restriction reversed the majority of these changes [6]. In a recent study with C57BL/6J mice, the expression profiles of liver tissues from eight-month-old and 32-month-old animals were compared [7]. Aging was accompanied by suppression of genes involved in the insulin growth factor-1/growth hormone pathways, carbohydrate metabolism and ATP biosynthesis, xenobiotic metabolism, and peroxisomal biogenesis. Finally, old animals exhibited an increase in the immune and inflammatory responses and an upregulation of genes associated with protein and amino acid glycosylation. Overall, liver from old mice exhibits a decrease in growth/proliferation pathways and metabolisms that would increase oxidative stress, and an increase in inflammatory and stress response genes. 

Several theories have been proposed for the age-associated alterations observed in the liver, and one of them is the somatic mutation theory of aging. This theory was followed by the DNA damage theory of aging [8], which proposes that the accumulation of genomic instability in cells of a tissue, particularly in the stem cell compartment of the tissue, will modify several genes that can eventually lead to either cancer, apoptosis, senescence, or abnormal tissue homeostasis. A body of correlative data supports this theory, but how much genetic instability contributes to overall aging is still in debate. Nonetheless, it is important to mention that even though DNA repair may not change with age, mutations may still accumulate with the number of cell division in a tissue due to unavoidable DNA replication errors. In a situation in which DNA repair declines with age, unrepaired DNA lesions may persist with harmful consequences to the cells with age. Unrepaired DNA lesions may not only affect the effectiveness of copying chromosomes during DNA replication, but also the transcription of damaged loci. Finally, a shift from error free to error-prone DNA repair pathways during aging may also contribute to the accumulation with age. In this paper, we will use the mouse model to describe what is known about DNA repair in the aging liver and its probable implication in the age-associated physiological modifications.

## 2. Mutation Rate in the Liver of Mice with Age

The mouse is a good biological tool that allows the analyses of different tissues with little limitation on the amount of biological materials available. Mice are economical compared to larger mammals, and there is a huge volume of literature on the physiology, behavior, and biochemistry of such rodents. Importantly, it is possible to modify the diet of mice or treat them with drugs to mimic specific diseases and/or to improve their health status. Finally, their genomics and genetics have been extensively studied to such a point that now there is a battery of transgenic and knockout mice which, to some extent, phenocopy important age-related diseases. Many mice with mutations in different DNA repair proteins are available. Importantly, at least four transgenic lines with the *lacI *and/or *LacZ *reporter genes have been intensively used to estimate the mutation frequency or rate in the genome of different tissues with age. One such transgenic line bears a lambda shuttle vector that carries a *lacI* target and an alpha *lacZ* reporter gene [9, 10]. Genomic DNA is isolated from the tissue under study, and the shuttle vector is recovered by exposing the DNA to lambda phage packaging extracts *in vitro*. Mutations in the* lacI* target gene that inactivate the repressor gene allow expression of the alpha *lacZ *reporter gene, resulting in blue mutant plaques. Sequencing of the DNA from these plaques not only allows the estimation of the mutation frequency, but it also points to the type of mutation providing insights into potential mechanisms [9, 10]. The *lacI* gene is highly sensitive to base substitution and frame shift mutations, as well as small deletions and insertions, making the transgene an ideal choice for recovery of spontaneous and induced mutations [11, 12]. The Big Blue mouse contains approximately 40 copies of the lambda shuttle vector stably integrated as a tandem array at a single position in chromosome 4 [12]. The MutaMouse contains the sequence of a phage carrying the *lacZ* gene integrated in a head-to-tail arrangement of approximately 40 copies located at a single insertion site in chromosome 3 [13, 14]. The technical difference in identifying mutations in these two mouse systems is that the Big Blue mouse model is based on forward mutations in the *lacI* reporter sequence derepressing the *lacZ* gene thereby yielding blue plaques as mutants. Thousands of plaques need to be examined. The Muta mouse is based on forward mutations in the *lacZ* reporter gene that can be easily selected because only mutants will generate plaques. Finally, the *lacZ *transgenic mice lines 30 and 60 bear a plasmid carrying the *lacZ* gene. Line 60 was found to have two integration sites, which were mapped to chromosomes 3 and 4. The plasmid integration site of line 30 is on chromosome 11. Each integration site in both transgenic lines has about ten to twenty plasmids per haploid genome [15]. Plasmids are rescued by excision with the restriction enzyme HindIII, followed by separation from mouse genomic DNA by the use of magnetic beads coated with the lacI repressor protein, which will bind the *lacI* sequence. The recuperated DNA is then self-ligated to obtain circular plasmids that are finally transferred into *Escherichia coli* C bacteria (harboring a deletion of its own *lacZ *gene) for sequence analyses [15, 16]. Mice of line 60 are appropriate transgenic animals for the study genome rearrangements in the aging liver [15, 17], and chromosomal translocations and deletions up to 66 megabases have been observed in the tissues of such mice [17]. Such chromosomal rearrangements cannot be detected using the phage-based reporter models (the MutaMouse and the Big Blue models).

Before describing the findings obtained with these transgenic mouse mutation detection systems, one needs to have an overview of liver growth and development. The following information was obtained from a paper published by Stuart and Glickman in 2000 [10]. The liver is regarded as a slowly renewing tissue. The liver from two-week-old CBA/C57BL mice contains approximately 0.8 × 10^8^ hepatocytes/cm^3^ of tissues. This number increases by 1.5-fold to approximately 1.2 × 10^8^ cells in three-month animals, then decreases slightly to 0.98 × 10^8^ and 0.88 × 10^8^ cells/cm^3^ at ages of 12 and 24, respectively. Cell proliferation decreases significantly (approximately 3.3-fold) in the liver of male mice from ages 2.5 to 32 months. Although the number of cells in the postnatal liver reaches a plateau, DNA synthesis continues at a reduced rate throughout adulthood, resulting in an age-related increase in mean polyploidy. Thus, mean DNA ploidy levels in mouse liver double from ages of one week to one month and thereafter increase steadily, doubling again by 24 months of age. This increase in liver polyploidy is accompanied by an increase in liver weight but not in cell number. From the age of two to three weeks, it has been reported that each liver cell in the mouse enters the mitotic cycle from one to six times (three on average), resulting in an eight-to-ten-fold increase in liver mass and about a threefold increase in the number of cells. Mature hepatocytes are fully differentiated, self-maintaining cells with low proliferative rate and low, if any, rate of cell elimination from the population during the life of the mouse. The liver cells in newborn mice are diploid but polyploidy levels increase in young animals [10]. The presence of advanced polyploidy in mammalian cells is generally considered an indication of terminal differentiation and senescence of cells [18]. As pointed out by Gupta in 2000 [18], although the existence of a liver stem cell is often debated, most experts agree that progenitor liver cells are activated in response to significant depletion of hepatocytes following exposure to hepatotoxins or carcinogens. It has been estimated that mouse hepatocytes have a turnover time of 480–620 days [10]. Thus, an adult mouse would have up to 30–40% of hepatocytes with more than one nucleus. Multinucleated hepatocytes are thought to develop in response to completion of DNA synthesis and mitosis but with failure of cell division or cytokinesis, which would normally generate daughter cells containing single nuclei [18]. The exact molecular mechanisms leading to polyploidy in the liver are still unknown. Recent evidence, however, suggested that key components of the insulin pathway (PI3K and Akt kinases) control cytokinesis failure events. For example, rats injected with insulin exhibit increased tetraploidization in the liver [19]. Interestingly, polyploid hepatocytes are under oxidative stress, as suggested by an antioxidant depletion, increased lipid peroxidation, and elevated 8-hydroxyguanine in nuclear DNA. Accordingly, introduction of oxidative injury significantly induced polyploidy in cultured hepatocytes freshly isolated from rat liver [20]. Liver repopulation assays (after partial hepatectomy) in rodents also show markedly decreased replication capacity in polyploid hepatocytes [20]. Thus, the diploid cells will be responsible for liver regeneration after a partial hepatectomy as they still retain their full proliferative potential unlike polyploidy cells. The presence of senescence-type changes in polyploidy hepatocytes is likely to affect normal liver homeostasis during aging. In addition, cell polyploidy is a potential source of aneuploidy, DNA rearrangements, and tumorigenesis. Indeed, chromosomal aberrations after partial hepatectomy increase with aged mice. Additional results based on the analyses of the *lacZ* reporter plasmid (transgenic mouse line 60) showed a rapid accumulation of genome rearrangements in the liver of old mice (27 months of age and older) [15]. On average, there was a two-to-four-fold increase in liver genome rearrangements involving the *lacZ* reporter gene over the life span of mice [15, 17]. The authors of these studies hypothesized that the increase in DNA rearrangements in the liver of old mice was due to oxidative stress [15].

The free radical theory of aging, first proposed by Harman in 1956 [21], has received a lot of attention over the years as indicated by the number of scientific reviews on antioxidant interventions in different animal models and human clinical trials. The mitochondrion has been identified as a major source of reactive oxygen species (ROS) and thus oxidative stress potentially contributing to the aging process, although several plasma membrane and cystosolic enzymes may also contribute to the increased intracellular pro-oxidant status observed during aging [22]. In the mitochondrial respiratory chain, electrons entering complexes I and II are transferred to complex III, then IV where they are combined with molecular oxygen and hydrogen to form H_2_O. Redox reactions at respiratory complexes I, III, and IV are coupled to the extrusion of protons from the mitochondrial matrix into the intermembrane space. The re-entry of protons into the matrix is coupled to the synthesis of ATP from ADP and P_i_. This oxidative phosphorylation is responsible for the vast majority of ATP production and oxygen consumption in most types of animal cells [23]. Up to 2% of oxygen used in this complex reaction undergoes monoelectronic reduction and results in the formation of superoxide anion and hydrogen peroxide, which can lead to the formation of the more toxic species hydroxyl radicals [24, 25]. Such reactive species can attack and modify genomic DNA. An important type of oxidative DNA lesion accumulating with age is 8-oxo-deoxyguanine [26]. If unrepaired, this adduct in genomic DNA may lead to a point mutation upon DNA replication. During DNA replication, 8-oxo-deoxyguanines present on either strand of DNA can mispair with adenosines and lead to G:C → T:A transversion mutations. A misincorporation of an 8-oxo-deoxyguanine as a substrate nucleotide can also lead to the same type of mutational pattern [27].

The Big Blue transgenic mouse detection system was used to examine the spontaneous point mutation frequency and pattern in the aging liver [9, 10, 12, 28]. The major conclusions from these studies are that the spontaneous mutation frequency increased with age in mouse liver, although no specific type of point mutation predominated (transversion or transition type of mutation) with age. The only exception was an increase in the frequency of GG → TT tandem-base mutations, which specifically increased with age in the liver. Although there was a lot of interindividual variations between old mice, overall the liver showed approximately two-to-four-fold increase in point mutation frequency from 10 to 30 months of age [28]. In contrast, the GG → TT tandem transversion mutation in the liver of Big Blue mice increased eight-fold in middle-aged animals (compared to young mice) and 20-fold by 25 months of age [12]. The GG → TT tandem-base mutation represented a mutational signature of an age-related change in older liver not seen in other tissues. The increased frequency of endogenous GG → TT tandem-base mutation pattern in the aging liver may be due to mutagenic acetaldehyde derivatives, which can result from lipid peroxidation reactions [12], as the liver is the major site for lipid metabolism in the body [2]. Notably, the mutation frequency and pattern (type of mutations) in other tissues, like brain or germline tissues for example, were different than in liver [9, 10, 12, 28]. This conclusion was also reached with *lacZ* transgenic mouse model [29].

In view of the low mutation frequency in the liver and the low levels of DNA rearrangements, several authors argued that such changes in the genetic material would not be sufficiently high to have an impact on liver function with age. It is possible, however, that the transgenic mouse mutation detection system is underestimating the number of persistent DNA lesions (or damage) that would affect DNA replication or transcription. They also miss mutations that only decrease gene activity, but which can still have an impact on aging. Finally, the transgenic reporter systems cannot detect mitotic recombination events that can lead to somatic mutations [30].

## 3. Levels of DNA Damage in the Mouse Aging Liver

As discussed above nucleic acids can be oxidized by ROS that are formed as natural byproducts of the mitochondrial metabolism of oxygen. In addition, organisms are constantly exposed to exogenous sources of ROS and environmental agents or pollutants that can damage DNA. The most common pollutants include car exhaust particles, cigarette smoke, UV and ionizing radiation, insecticides, and pesticides, among others. The most characterized mutagenic DNA lesion appearing due to endogenous metabolism is 8-oxo-deoxyguanine. The presence of this lesion in the DNA results from either the direct oxidation of guanine bases in the DNA or as a consequence of incorporation of 8-oxo-7, 8-dihydro-2′-deoxyguanosine 5′-triphosphate (8-oxodGTP) during replication [31]. Several publications have indicated that mice accumulate 8-oxo-deoxyguanine lesions in their liver with age by approximately 1.8-to-2.8-fold [31–34]. Concomitant with the increase in 8-oxo-deoxyguanine lesions, persistant single-strand breaks have also been described in the liver of aging mice [32]. This is, however, still debated as there are several reports indicating no increase in 8-oxo-deoxyguanine lesions or single-strand breaks in the liver of old rodents compared to young animals [35, 36]. It has been suggested that the discrepancies observed in the literature are due to artifactual DNA oxidation, which arise during the isolation and analysis of the DNA samples [34], and a consortium of laboratories (the European Standards Committee on Oxidative DNA Damage, ESCODD) was established to look into these methodological aspects [37]. 

Formamidopyrimidines such as 2,6-diamino-4-hydroxy-5-formamidopyrimidine and 4,6-diamino-5-formamidopyrimidine are also DNA lesions occurring due to oxidative stress related to mitochondrial metabolism [31], which can be detected in liver DNA at levels comparable to or even higher than those of 8-oxo-deoxyguanine in six-month-old animals [38]. In contrast to 8-oxo-deoxyguanine lesions, however, formamidopyrimidine lesions did not increase in the liver of old mice [31]. It has been estimated that 8-oxo-deoxyguanine makes up approximately 5% of all oxidative lesions [39]. Although the steady-state levels of 8-oxo-deoxyguanine would seem relatively small in the genome of rodent cells, the accumulation of 8-oxo-deoxyguanine in specific sites in the genome is expected to be highly relevant for aging. 

Telomeres are DNA structures composed of TTAGGG repeats of several kilobases (thus G rich sequence) required for the maintenance of chromosomal ends. They protect chromosomes from end-to-end fusion, recombination, and degradation. It is now well accepted that an increase in chromosome instability may be associated with loss of telomeric repeats and cellular senescence [40, 41]. Senescent cells are often oblivious to external growth stimuli and may affect the rejuvenation or normal homeostasis of tissues with age. Thus, due to its high guanine content, the telomeres are hot spots for oxidative damage, at least in human cells [42]. More importantly, 50% of single-strand breaks remain unrepaired in telomeres unlike the same kind of damage in other part of the genome [42]. Embryonic fibroblasts from mice lacking the DNA glycosylase OGG1, which removes 8-oxo-deoxyguanine, showed telomere shortening and increased sister chromatid exchange, indicating an important role of this lesion in telomere dysfunction [43]. Finally, it has been reported that a single 8-oxo-deoxyguanine lesion in a defined telomeric substrate reduced the percentage of bound TRF1 and TRF2 proteins by at least 50%, compared with undamaged telomeric DNA [44]. Thus, oxidative DNA damage may also exert deleterious effects on telomeres by disrupting the association of telomere maintenance proteins TRF1 and TRF2 [44]. “Uncapped” telomeric 3′-overhang sequence TTAGGG will induce a senescent phenotype in cultured human fibroblasts [45]. Despite the observation of telomere attrition in OGG1-deficient mice, such mice do not exhibit a premature aging syndrome. These results suggest that other unknown processes are at play during aging or that the extent of telomere dysfunction is not as high in tissues as one would see after several generation in mice lacking the enzyme telomerase required for telomere maintenance [46]. 

A recent study has indicated a good correlation between the presence of senescent cells in the liver of mice (detected on cryosections with the senescence associated *β*-galactosidase) and the presence of DNA breaks detected by immunohistochemistry with an antibody against the double-strand DNA break marker *γ*-H2AX [47]. The vast majority of *γ*-H2AX foci-positive cells in the liver were hepatocytes as judged by morphological criteria. The frequency of *γ*-H2AX foci-positive cells increased significantly with age by twofold in the liver of mice. More precisely, *γ*-H2AX foci-positive cells increased from 17% of the hepatocytes in the liver of 12-month-old mice to 34% of hepatocytes in 42-month-old animals [47]. At all ages, however, the frequencies of foci-positive hepatocytes in the periportal areas were lower than in the centrilobular and intermediate areas of the lobes. Thus, some regions of the mouse liver are more likely to accumulate DNA breaks with age. It is worth mentioning that not all *γ*-H2AX foci-positive cells in the liver of mice are also positive for senescence-associated *β*-galactosidase [47]. The potential problems with *γ*-H2AX foci-positive cells are that they can die by apoptosis or accumulate carcinogenic lesions. Dead cells must be replaced by a constant renewing of stem cells. If stem cells are senescent and do not respond to environmental stimuli to divide, the accumulation of senescent cells with age will eventually affect liver rejuvenation and the normal homeostasis of this tissue.

There is evidence for an increase susceptibility to DNA damage in the liver from exogenous toxic agents (including oxidative stressors) with age. For example, the number of 8-oxo-deoxyguanine lesions in hepatic DNA of 14-month-old mice treated with carbon tetrachloride, known to generate ROS in the mouse liver, was significantly higher than that of the two-month-old animals treated with the same chemical [33]. Similarly, DNA lesions (mainly single-strand breaks) induced by the oxidizing agent nitroquinoline-N-oxide increased with age in the liver of mice [32]. Hepatic DNA from older mice are also more prone to alkylating damage than younger animals. In one report, liver DNA in old (29 months) mice accumulated 50% more damage when treated with the alkylating agent N-methyl-N-nitrosourea (MNU) than young (nine months) animals treated with the same amount of the drug (doses normalized on weight) [48]. N-nitroso compounds are widely disseminated in the environment and are important food contaminants. A major mutagenic DNA lesion produced by MNU in the liver of mice is the 7-methylguanine. Interestingly, the same study with MNU also revealed age-related changes in chromatin composition or structure that make some genomic sequences more accessible to alkylating agents in liver tissue of older animals [48]. Liver chromatin can be fractionated into nuclease-soluble, low-salt, high-salt, and nuclear matrix fractions. All fractions of liver chromatin from young mice (nine to eleven months) were equally modified by MNU. In contrast, nuclease-sensitive regions of liver chromosomes from old mice (28-29 months) were preferentially alkylated by MNU over bulk chromatin and nuclear matrices [48]. With regards to liver chromatin, it is worth mentioning that there is an age-dependent increase in the amount of DNA protein cross-links in some strains of short-lived mice compared to long-lived mouse strains [49]. The reason for this difference has yet to be determined.

Finally, the metabolism of procarcinogens in the liver will impact on the levels of DNA damage with age. For example, the genotoxicity of the environmental pollutant polycyclic aromatic hydrocarbon benzo[*α*]pyrene is dependent on its metabolic activation by liver P450 cytochrome monooxygenase enzymes [50]. The reactive metabolite binds predominantly to guanines in the DNA. Such DNA adducts are mainly repaired by the nucleotide excision repair pathway. Interestingly, the formation of DNA adducts by benzo[*α*]pyrene was decreased by approximately threefold in the liver of 18-month-old animals compared to two-month-old mice [50]. This decrease is concomitant with the reduced total microsomal P450 content observed in rodent liver tissue with age [51].

## 4. DNA Repair Enzymes in the Liver of Old Mice

DNA repair is required for genome stability. If lesions in DNA were not repaired, they would accumulate to high levels, incompatible with normal conditions of life. Cells have evolved a complex network of repair systems for the removal of DNA damage. If unrepaired, DNA lesions can lead to DNA polymerase arrest, which can result in double-strand breaks upon DNA replication collapse or breaks at regions of the genome that are actively transcribed and mutations. Both strand breaks and mutations accumulate with age in several model organisms, indicating an important role for DNA repair in preventing age-associated genomic instability. Because the chemical nature of the DNA lesions is varied, each type or class of DNA modification is repaired by a specific repair pathway. Small base modification, such as 8-oxo-deoxyguanine, many alkylated bases, and single-strand breaks are mainly recognized by enzymes of the base excision repair pathway (BER). Bulky covalent lesions, such as those induced by UV and several carcinogens, are repaired by the nucleotide excision repair pathway (NER). Double-strand breaks and interstrand DNA cross-links, on the other hand, require a specialized repair pathway and are repaired by non-homologous endjoining or by homologous recombination. It is noteworthy, however, that while these repair pathways have been characterized independently, *in vivo* there is substantial crosstalk among them. Some proteins involved NER may, in some cases, also participate in the repair for certain oxidative lesions, mostly via protein interactions with canonical BER proteins.

### 4.1. Base Excision Repair in the Liver of Mice

Base excision repair (BER) functions by glycosylase-initiated removal of a damaged base followed by incision of the DNA backbone, synthesis of new DNA, cleaning up of the 3′ and 5′ ends, and ligation [52]. Various DNA glycosylases recognizing specific types of damage initiate BER. Incision of the phosphate backbone is accomplished by an AP endonuclease, APE (HAP1, APEX, REF1). In the nucleus, the DNA synthesis step in the BER pathway is carried out by DNA polymerase *β* (*β*-pol) [53]. It is estimated that 70–90% of all BER takes place via the replacement of a single nucleotide by *β*-pol, short-patch pathway [54]. In a minor subpathway termed long-patch BER, *β*-pol is believed to initiate repair synthesis and can replace up to six nucleotides [55]. The long-patch BER is also DNA polymerase *δ*/PCNA- and FEN1-dependent [56, 57]. Poly(ADP-ribose) polymerase 1 (PARP1), functioning as DNA nick-sensor, will regulate the activity of *β*-pol during long-patch BER [58]. In 2002, Cabelof and colleagues [52] reported their analyses of uracil-initiated short-patch BER in liver extracts of C57BL/6 mice at different ages. The G:U mismatch is estimated to be responsible for 70–90% of all BER [54]. Nuclear extracts from the liver of 22–26 month-old mice showed a 50–75% decrease in the repair of a DNA duplex containing G:U mismatch compared to the liver of young four-to six-months-old animals [52]. A similar decrease in the liver of old mice was also obtained with a DNA duplex containing an 8-oxo-deoxyguanine, although no changes in OGG1 activity in the nucleus of mouse liver with age had been observed previously [59]. Interestingly, they found a decrease in *β*-pol protein levels in the liver of old C57BL/6 mice compared to young mice. The group also examined the* lacI* mutation frequency in response to the alkylating agent dimethyl sulfate in their young and old animals. The mutation frequency did not significantly increase in young animals whereas identical exposure in aged animals resulted in a fivefold increase in mutation frequency. Because dimethyl sulfate induces DNA damage processed by the BER pathway, the authors suggested that the increased mutagenicity of dimethyl sulfate with age was related to a decline in BER capacity that occurred in the liver of aged mice [52]. The predominant lesion induced by dimethyl sulfate is N^7^-methylguanine, which is efficiently repaired by BER. Because the glycosylase activity for N^7^-methylguanine does not appear to decrease during aging [60], it is likely that the decrease in BER observed in old C57BL/6 mice is due to a decrease in *β*-pol activity. The following year, Intano and colleagues [61] also observed, in two different mouse strains (outbred CD1 and hybrid B_6_D_2_F_1_ mice), a 50% decrease in G:U mismatch repair in nuclear extract from the liver of aged mice. They were unable, however, to see a difference in *β*-pol protein levels in the liver of young and old mice (3-, 18-, and 28-month-old mice). The levels of other proteins important for BER of G:U mismatch such as ligase I and III, XRCC1, APE/REF-1 were not changed with age [61]. The difference in *β*-pol protein levels in C57BL/6 mice with age could be mouse strain-specific, or it could even be due to differences in husbandry conditions [62].

Removal of 8-oxo-deoxyguanine in chromosomes is primarely initiated by the 8-oxoguanine DNA glycosylase 1 (OGG1) or, to a lesser extent, Nei endonuclease VIII-like 1 (NEIL1) repair enzymes [31]. In the nucleus, the expression of OGG1 and NEIL1, at least at the mRNA level, does not change with age in the liver of C57BL/6 mice. Similarly, 8-oxo-deoxyguanine glycosylase activity was not significantly changed with age in mouse or rat liver [59, 63]. In the nucleotide pool, 8-oxodGTP is generated by the oxidation of 2′-deoxyguanosine 5′-triphosphate (dGTP). One of the nudix enzymes (nucleoside diphosphate-linked moiety X)-type motif 1 (Nudt1, also called MTH1) degrades 8-oxodGTP to 8-oxodGMP, thereby preventing its incorporation into chromosomes during DNA replication [31]. The mRNA levels of Nudt1 do not change with age in mouse liver. Intriguingly, the 8-oxodGTPase activity in the liver significantly decreases by approximately 30% from six to twelve months compared to two-to-three-month-old mice. It then increases back to levels observed in the liver of two-three-month-old animals when mice reach the age of 25 months [31], although the levels of 8-oxo-deoxyguanine lesions were still higher in the liver of 25-month-old animals [31]. The authors suggested that the increase in 8-oxodGTPase activity observed in older animals could be an adaptive response to an increase in endogenous ROS production that can potentially overwhelm the antioxidant systems of aged hepatocytes. Food consumption per body weight is also higher in two-to-three-month-old mice compared to six-to-twelve-month olds, potentially contributing to higher endogenous metabolic ROS in young animals. Appropriate experiments are warranted to confirm this hypothesis. Overall, oxidative DNA damage increases in the liver of mice with age concomitantly with a decline in BER. Knockout of several BER enzymes has been generated in mice over the years. A comprehensive review on this subject has been already published by Xu and colleagues and will not be discussed here [62].

### 4.2. Nucleotide Excision Repair in the Liver of Mice

The nucleotide excision repair (NER) is the main DNA repair system for the removal of bulky and helix distorting lesions in DNA. The NER pathway is composed of a multiprotein complex that removes an oligonucleotide containing the lesion. The 5′-incision, which is carried out by an endonuclease complex containing the excision repair cross-complementing rodent repair deficiency, complementation group 1 (ERCC1) protein, is the first step in the excision of the nucleotide and can be considered as the rate-limiting step of the NER process [31]. The mRNA levels of Ercc1 do not change with age in the liver of mice [31]. One study, however, has indicated a significant decrease in NER activities in hepatocytes from 24-month-old rats compared to hepatocytes from six-month-old rats [64]. This difference with age, though, was specific to certain regions of the genome. Accordingly, there are age-related changes in the structure and function of the chromatin in different regions of the genome, which may affect the efficiency of DNA repair [65, 66].

The expression of several proteins involved in the NER pathway was found to decrease with age in the skin of mice [67], although the same has not been observed in the mouse liver tissue. Nonetheless, knockout of several NER proteins has been generated in mice over the years [68], recapitulating some of the human segmental progeroid syndromes like Xeroderma pigmentosum, Cockayne syndrome, or trichothiodystrophy [68]. Several of these mice exhibit, in addition to an accumulation of mutations in their liver tissues, transcriptional changes related to metabolic abnormalities or phenotypes associated with aging (discussed below).

### 4.3. Repair of Double-Strand Breaks in the Liver of Mice

Two major repair pathways are involved in the repair of double-strand breaks in order to preserve genomic integrity: the non-homologous end joining (NHEJ) and homologous recombination (HR). The NHEJ system repairs double-strand breaks in the DNA by joining free ends together without the aid of a homologous template and occurs during the G_1_ and S phases of the cell cycle. This reaction is therefore prone to errors during repair. The major proteins involved in this pathway include Ku70, Ku80, DNA-PK_CS_, Artemis, XRCC4, DNA Ligase IV (LIG4), WRN, and the XRCC4-like factor [69]. Ku70 and Ku80 form a heterodimer that binds to DNA ends and together with a PI-3 kinase catalytic subunit, DNA-PK_CS_, forms a holoenzyme referred to as DNA-PK (DNA-dependent-protein kinase). Artemis opens hairpins and processes overhangs in a complex with DNA-PK_CS_, thereby generating substrates that are ligated by the XRCC-LIG4 heterodimer in a complex with the XRCC4-like factor [69]. Finally, the exonuclease/DNA helicase WRN protein cooperates with the XRCC4-DNA ligase IV complex during end-processing [70]. Importantly, many proteins of the NHEJ reaction are required for the maintenance of the telomeric ends of chromosomes, including Ku70, Ku80, DNA-PK_CS_, and WRN [71]. These proteins will impact telomere length maintenance and will suppress telomere fusions that could eventually lead to chromosome rearrangements [69, 71].

In the HR reaction, the sister chromatid or homologous chromosome is used as a template to repair the broken DNA. Thus, HR is largely restricted to late G2 and M phases of the cell cycle, when these are available after DNA replication [71]. The first step in HR is processing of the double-strand break by a nuclease to generate 3′ single-stranded DNA tails, which are coated with the single-strand binding protein RPA. The MRN complex, composed of the proteins MRE11, RAD50, and NBS1, is a candidate for this nuclease, although other nucleases are likely involved as well. The RAD51 protein, assisted by a number of factors including RAD52, RAD54, BRCA2, and the RAD51 paralogs (XRCC2, XRCC3, RAD51B, RAD51C, and RAD51D), forms a nucleoprotein complex with the DNA and directs the 3′ single-stranded DNA tails to search out, invade, and pair with undamaged homologous sequences. DNA polymerases then carry out repair using the intact DNA as a template. The processes of DNA strand exchange and extension generate Holliday junctions, structures in which two double-stranded DNA duplexes are intertwined. The final step of the reaction is the resolution of the Holliday junction structures with specific nuclease enzymes [72]. Interestingly, several enzymes involved in NER also participate in different steps of the HR reaction. The proteins mentioned here form only a partial list, which is not intended to be complete, as there are several excellent reviews on the molecular events in HR.

Any defect in the double-strand breaks repair pathway is likely to lead to gross chromosomal rearrangements. There are indirect evidences that such repair systems might be affected in mouse liver with age. It is now well recognized that persistent double-strand breaks and gross chromosomal rearrangements increase in the liver of aged mice [15, 73]. The NEHJ function has been shown to decline with age in the brain of rats [74, 75]. Liver from old rats, however, does not show a decrease in Ku70, Ku80, DNA-PK_CS_ protein levels or activities compared to young rats [76]. There is little information for mouse liver in the literature. Based on microarray expression data, Hoeijmakers and colleagues [7] observed a twofold increase in mRNA levels for RAD51 and RAD51-like 1 proteins in the liver of 32-month-old mice compared to two-month-old animals. Han and colleagues [77] found RAD21 mRNA to be elevated in the liver of old mice. RAD21 is also involved in the repair of double-strand breaks [78]. Such increase in RAD51, RAD51-like 1, and RAD21 mRNA levels may reflect a response to the higher levels of double-strand breaks observed in hepatocytes of older mice [73]. One important limitation of microarray analyses is the absence of information on the actual protein levels in tissues under study. It is thus unknown at present whether the enzymatic activities related to HR (or to the NHEJ) in the mouse liver change with age.

## 5. Mitochondrial DNA in the Mouse Aging Liver

The mitochondrial theory of ageing is based on the assumption that ROS or other free radicals generated as byproducts of the mitochondrial electron transport chain during the life span of an organism damage proteins, lipids, and the mitochondrial DNA, which are closely positioned to the sites of ROS generation in the electron transport chain [25]. The mitochondrial genome is a circular molecule of approximately 16 s and codes only for components of the oxidative phosphorylation system (13 proteins in addition to 22 tRNAs and 2 rRNAs). A single hepatocyte may contain up to 25,000 copies of mitochondrial DNA (four to ten copies per mitochondrion), which are continuously turned over along with mitochondria, independently of the cell cycle [79, 80]. Oxidative damage is believed to play a substantial role in mitochondrial mutagenesis [45] because the majority of mitochondrial mutations are GC to AT transitions, which are signature mutations for oxidized cytosines [80]. Mitochondria possess a functional BER mechanism, some activities indicative of double-strand break repair [81], and recently mismatch repair activities have been identified in human cells and rat liver mitochondria [82, 83]. On the other hand, NER has not been detected in mitochondria [84]. The Cockayne Syndrome group B gene product (CSB), which is involved in NER and BER in the nucleus, has recently been found in human mitochondria, where it is believed to only assist BER reactions through protein interactions [85].

One study reported that most mitochondrial DNA in the liver of 22-month-old mice carry multiple point mutations, a significant increase compared to two-month-old animals. The mutation frequency in the liver of old mice ranged from 0.1 to 2.4%, which exceeds the mutation frequencies observed for nuclear DNA by approximately 1000-fold [86]. This observation is not, however, surprising, as the levels of oxidized DNA lesions are significantly higher in the mitochondrial DNA when compared to the nuclear DNA in rat and mouse liver [34, 87]. As in the nuclear DNA, oxidative DNA damage can lead to mutations but also to deletions. Indeed, the frequency of multiple deletions in mouse liver also increases significantly with age [88, 89]. The exact reason for the increase in deletions in the aging liver is yet unknown, but an important clue to the mechanism was given by the observation that the deleted regions in the mitochondrial DNA of old mice are flanked by small repeats [89]. Paradoxically, BER from mitochondrial extracts increases with age in the liver of mice and rats [59, 63], in contrast to data showing an increase in oxidative lesions in mitochondrial DNA with age [86]. This contradiction may have been resolved when it was found that although the total 8-oxoguanine-DNA glycosylase activity is higher in mitochondrial extracts from the liver of old mice, a large fraction of the enzymes is stuck to the membrane in the precursor form, which could not be translocated to and processed in the mitochondrial matrix. A similar phenomenon was observed with the mitochondrial uracil-DNA glycosylase responsible for the repair of mutagenic uracil in the DNA, suggesting a net decrease in repair with age [90].

DNA polymerase *γ* is the only known DNA polymerase in mitochondria, involved both replication and repair processes. This protein is encoded by a nuclear gene. Two groups have independently generated mice expressing a proof-reading-deficient DNA polymerase *γ* [91, 92]. These mice accumulate mitochondrial DNA mutations and deletions and exhibit a premature aging phenotype with a reduced life span. Interestingly, such mice do not exhibit increased markers of oxidative stress (including oxidized DNA damage) in the liver with age [92, 93]. The only age-related phenotype observed in the liver of proof-reading-deficient DNA polymerase *γ* mutant mice was extramedullary haematopoisis in the liver of six-month-old animals [91], while this phenotype generally occurs much later in normal mice [94]. It is believed that the accumulation of mitochondrial mutations in such mice is causing a decrease in functional proteins of mitochondrial electron transport chain [95]. This, in turn, impacted on energy production, cell proliferation and led to increased apoptosis in the liver tissue potentially affecting liver homoeostasis [91–93, 95]. Electron microscopical analyses of the mitochondria from the DNA polymerase *γ* mutant mice should give important information on the morphology and function of these organelles in the liver. Finally, the levels of telomeric rearrangement or attrition in such mice would also contribute to important information on the impact of mitochondrially generated ROS on telomere stability in aging mice.

## 6. DNA Repair Enzymes and Metabolic Syndromes

A major question that still remains is whether DNA repair deficiency leads to accumulation of enough mutations in the liver (or any other tissue) to cause aging or any age-associated diseases. Most DNA repair encoding genes have been mutated or deleted through genetic manipulation of embryonic stem cells to understand their functions and impact on aging. The goal of this section is to describe some of these mouse models of aging that we think are giving valuable information in the field of liver aging.

### 6.1. Base Excision Repair

The genetic ablation of XRCC1, APE/REF-1, *β*-pol, and DNA ligase III in mice is embryonic lethal [96–99], while the absence of 8-oxoguanine DNA glycosylase 1 (OGG1), endonuclease III homolog (mNTH1), or 8-oxodGTPase (MTH1) results in animals appearing normal, with no gross abnormality in the liver. They do accumulate premutagenic lesions, 8-oxoguanine DNA, and are prone to hepatocarcinoma in the presence of damaging agents (causing oxidative stress) [100–105], but no aging phenotype has been observed. PARP1-deficient mice, in turn, exhibit increased frequency of deletions and spontaneous liver tumors compared to wild-type animals [106]. In contrast, a knockout mouse model of the NEIL1 DNA glycosylase in mouse can result in a severe metabolic disorder [107]. Metabolic syndrome afflicts up to one half of Western population and is considered an age-related, pro-inflammatory lipidic disorder [108]. This phenotype, however, has not been reproduced in NEIL1-deficient mice from other laboratories and could be due to the genetic background of the mouse colony used in the published study [107]. Although OGG1 and NTH1 enzymatic reactions represent the bulk of DNA glycosylase activities in mammalian cells for repairing oxidized bases, NEIL1 is part of a back-up system for repairing ROS-induced lesions in mammalian mitochondria [38] and cells. Importantly, NEIL1 protein, unlike OGG1 and NTH1, is able to excise base lesions from single-strand DNA regions suggesting a preferential involvement of this enzyme in repair during transcription [109]. Whether a specific subset of genes involved in glucose and lipid metabolisms is more prone to the deleterious effect of NEIL1 loss of function in the liver of mice awaits further evidence.

### 6.2. Nucleotide Excision Repair

Two subpathways are recognized for the NER, which differ in the lesion recognition mechanisms. The global genome NER is involved in the removal of distorting lesions anywhere in the genome, while the transcription-coupled NER eliminates distorting DNA damage that blocks transcription [110]. As mentioned earlier, several mouse models with mutations in different NER genes were generated to phenocopy different progeroid syndromes [68]. These models include the XPA, CSB, ERCC1 knockout mice and the XPD point mutation animals [110]. Briefly, the XPA knockout mice are completely deficient for both global genome NER and transcription-coupled NER, but can repair some transcription-blocking lesions, including oxidative DNA damages. The CSB protein is thought to be involved in displacing RNA polymerase stalled by a DNA lesion and recruiting the NER (and probably the BER) machinery to sites of DNA lesions. CSB knockout mice are deficient in transcription-coupled NER and have lower BER activity of oxidative DNA damage, while their global genome NER capacity remains intact. Such mice show mild aging features including reduced growth and mild neurologic dysfunction [111]. Complete inactivation of the XPD helicase is not viable in mice, due to its essential transcription initiation function as a component of the Transcription Factor IIH complex. There is an XPD mouse model, however, that contains the same point mutation found in a trichothiodystrophy patient, which shows many hallmarks of trichothiodystrophy, including premature aging features [68]. This mutant XPD protein causes a partial defect in both global genome and transcription coupled NER. The ERCC1-XPF complex forms an endonuclease required for the 5′-incision to remove the damage-containing oligonucleotide during NER, but also essential for interstrand crosslink repair. ERCC1 knockout mice are deficient in global genome NER, transcription-coupled NER, and interstrand crosslink repair. These mice show a severe phenotype including retarded growth, progressive neurological abnormalities, kyphosis, a short life span of about three to four weeks, and liver dysfunction [112]. At the cellular level, an ERCC1 defect leads to accelerated nuclear polyploidization. The combination of a knockout allele with a truncated ERCC1 allele, resulting in a protein lacking the last seven amino acids, delays the onset of the premature aging phenotype and extends the maximal life span to about six months [113].

XPA, CSB, ERCC1 knockout mice and the mutant XPD animals were crossed to mice harboring *lacZ*-reporter genes, and mutation frequency was estimated in the liver of these mice by Dollé and colleagues [110]. XPA deficiency resulted mainly in one-base pair deletions in young mice, but in older mice the mutation spectrum changed to an increase in G:C → T:A transversions, which are characteristic of oxidative DNA damage, indicating that an increase in oxidative DNA damage is an important event in age-related mutagenesis in the liver. ERCC1 mutant mice (expressing the truncated protein), with their short life span of six months and severe symptoms of premature aging, displayed an even faster *lacZ*-mutant accumulation in the liver. Mutations included mostly genome rearrangements [110]. In contrast, CSB knockout mice and the XPD point mutation animals did not reveal an elevated *lacZ* mutation frequency. The authors concluded that shortened life span in mice with defects in transcription-coupled repair did not depend upon increased mutation accumulation [110]. Interestingly, global gene expression analyses using microarrays revealed that the same mutated XPD mice exhibited increased apoptosis that exceeded cell renewal in their liver [114]. Appropriate histochemical analyses confirmed the microarray data. In addition, these mice displayed major metabolic changes with regards to lipid metabolism (abnormal lipofuscin accumulation in hepatocytes) and a downregulation of the Insulin Growth Factor 1 (a known regulator of life span) signaling in their liver [114, 115]. In the case of ERCC1 knockout mice, metabolic and gene expression profiling also revealed altered lipid and energy metabolism, transition to ketosis, increased liver cell death, and attenuation of liver functions [7, 116]. Finally, these mice also displayed downregulation of the Insulin-like Growth Factor 1 signaling in their liver [116].

A double knockout of XPA and CSB genes in mice resulted in a complete inactivation of NER leading to a phenotype that reliably phenocopied the human progeroid Cockayne syndrome [117]. Mouse liver transcriptome analysis and several physiological endpoints revealed systemic suppression of the growth hormone/insulin-like growth factor 1 somatotroph axis, changes in oxidative metabolism with increased antioxidant responses, and hypoglycemia together with hepatic glycogen and fat accumulation [117]. Importantly, wild-type mice exposed to a low dose of chronic genotoxic stress recapitulated this response. Altogether, these results suggest that even though mutation rates may not be tremendously high in these mice, impaired transcription of genes containing DNA lesions would still contribute to aging due to the intimate relationship between NER and transcription in the liver of NER-deficient mice [69].

### 6.3. Double-Strand Break Repair

There is a lot more information regarding mice deficient in proteins of the NHEJ repair pathway and aging liver than those lacking proteins of the HR pathway [69, 71]. DNA-PK_CS_-deficient mice develop hepatitis with very high frequencies (66% of all mutant mice examined) [118], although no other liver anomalies were reported. Ku80-deficient mice exhibit signs of premature aging in several organs, and their livers displayed micro abscesses (acute response with neutrophils) and granolamatous inflammation (chronic response with mononuclear cells), as well as nodular hyperplasia [119]. Moreover, Ku80-deficient mice harboring the *lacZ*-reporter gene displayed significantly higher numbers of genome rearrangements in liver compared to wild type animals [120]. The liver of such animals also exhibited substantial increase in persistent *γ*-H2AX DNA damage foci as compared to wild-type liver. On the other hand, little information has been published on the liver of Ku70-deficient mice, and only one report suggests that Ku70-deficient mice treated with an alkylating agent will rapidly develop hepatocellular carcinomas [121].

The WRN protein is a member of the RecQ helicase family that also contains a functional 3′-5′ exonuclease activity. It is involved not only in the NEHJ repair pathway but also in HR, in telomere maintenance, and potentially in BER pathways [122]. Complete functional inactivation of the WRN protein in human leads to the premature aging disorder Werner syndrome. The first mouse WRN mutant model to be described in the literature lacked part of its DNA helicase domain [123]. These mice synthesize a stable protein with no DNA helicase activity. Originally, these WRN mutant mice (largely in the outbred Black Swiss genetic background) showed almost no phenotype after one year of age, prompting several researchers to infer that these mice did not age prematurely [71]. This conclusion was reinforced by the description of a WRN null mouse that showed no age-associated phenotype [124]; these animals would show metabolic anomalies, including diffuse fatty infiltration of the liver, only if fed a diabetogenic diet [125]. However, WRN null mice crossed into telomerase knockout background exhibited metabolic anomalies and severe premature aging concomitantly with telomere attrition after several generations, stressing the importance of WRN functions at the telomeres [126, 127].

Realizing that the genetic background may have profound impact on the phenotype of mutant mice, the Lebel laboratory backcrossed the WRN helicase mutant outbred mice onto the pure C57BL/6N strain for twelve generations. These C57BL/6N WRN helicase mutant mice did not severely age prematurely, but displayed phenotypes associated with human Werner syndrome. They exhibit an abnormal hyaluronic acid excretion, premature visceral obesity, hypertriglyceridemia, insulin-resistant type 2 diabetes and associated cardiovascular diseases, higher ROS levels in several tissues, increased genomic instability, and cancer incidence resulting in a 10–15% decreased mean life span [128]. Importantly, WRN helicase mutant mice exhibit a severe defenestration of the liver sinusoidal endothelial cells at seven to nine months of age [129], which is not observed in wild-type animals until the age of two years. Interestingly, this morphological change is reversed by vitamin C or resveratrol treatments [129, 130], indicating a causative role for oxidative stress. In addition to morphological changes, WRN helicase mutant mice exhibit liver steatosis, increased lipid peroxidation, and oxidative DNA damage. Furthermore, these mutant mice displayed increased phosphorylation activities of kinases normally responding to oxidative stress [129]. Liver tissue from WRN helicase mutant mice also exhibited alteration in the expression of genes involved in caloric restriction [131], glutathione and xenobiotic metabolism by cytochrome P450 pathways. These results can be interpreted as a transcriptional response to the elevated oxidative stress in the mutant mice. These mutant mice also showed increased expression of genes involved in inflammation, indicating an inflammatory response in their liver [129, 130]. The observed alteration of the global gene expression profile in the liver of these mutant mice suggests that WRN protein may affect promoters of specific subset of genes directly [132] or indirectly. Finally, WRN helicase mutant mice exhibit increased rate of point mutations in liver mitochondrial DNA [129, 130]. Preliminary electron microscopy of liver tissue from helicase deficient WRN mutant mice has indicated a decrease in the number of mitochondria and altered morphology of these organelles compared to liver cells from age-matched wild-type animals [133], a phenotype reminiscent of ageing liver [134–136]. Concomitantly, WRN helicase mutant mice exhibited a decrease in ATP production [129]. Additional experiments will be required to determine whether the mitochondrial dysfunction is secondary to the dyslipidemia or diabetes seen in these mice [128, 129] or if it constitutes a primary event, which could be responsible for the premature aging phenotype.

## 7. Conclusions

Aging is a pleiotropic and stochastic complex process that is heavily influenced by genetic, epigenetic, and environmental factors. This is particularly relevant for the liver, which is a major organ regulating whole body homeostasis. The liver is central to glucose and lipid metabolism as well as steroid hormone biosynthesis and degradation. Importantly, it is the main detoxifying organ with regards to drugs or potentially toxic chemicals found in food. It has become clear, using mouse models as biological tools, that genetic instability in the form of gross DNA rearrangements or point mutations accumulate in the liver with age. DNA lesions, such as oxidized bases or persistent breaks, also increase with age and correlate well with the presence of senescent hepatocytes. The level of DNA damage and/or mutation can be affected by changes in carcinogen activation (during detoxification steps of procarcinogenic substances), decreased ability to repair DNA, or a combination of these factors [52]. ROS tissue level (exogenous or endogenous generated during normal metabolism) impacts on liver functions, and is intimately linked to most if not all age-associated diseases. Mitochondrial dysfunction is one major source of endogenous ROS during aging. Whether increased ROS is causative or a consequence of aging is still a subject of intense debate, especially in light of the observation that mitochondrial DNA polymerase *γ* mutant mice age prematurely without increasing ROS levels [92, 93] in different tissues including the liver. It is clear, however, that mitochondrial dysfunction due to accumulation of either mitochondrial DNA deletions or point mutations will affect energy balance in cells and, by the same token, the renewal of the stem cell compartment of a tissue or simply by altering highly metabolically active cells like the hepatocytes.

Telomere attrition is also associated with aging, although similarly to what was discussed for ROS, whether telomere shortening is causative or a consequence of aging is still a subject of debate. Considering the fact that the telomeric sequence is G rich, increased ROS will affect binding of telomere specific proteins and potentially induce a telomeric-dependent senescence pathway in cells, which again will impinge on liver homeostasis at the cellular level. In this context, telomeres would act as an “ROS sensor” in the cell gauging the levels of DNA damage in the liver. Causative or not, ROS levels and structural changes at the telomere will likely exacerbate the aging phenotype or dysfunction of a tissue. 

The *lacI/lacZ* reporter gene mouse models have taught us that different tissues exhibit different mutation rates with age. Specific DNA repair pathways have been shown to decline with age, depending on the tissues. Except for the BER pathway, few studies have shown decline of other DNA repair pathways or repair enzymes in the mouse aging liver. As several DNA repair enzymes are posttranslationally modified upon DNA damage (thus altering their activities), appropriate experiments are warranted to follow such posttranslational changes at the protein levels in the liver of aging mice. Noteworthy, the genetic background of the mice under study and the husbandry conditions (including diet) will also impact on the phenotypes. Thus, depending on the stress imposed on mice, the severity of the phenotype will vary. Nevertheless, the control of ROS levels, structural changes at the telomere, DNA damage and mutation rate, mitochondrial dysfunction will ultimately impact on health, and such processes underline the complexity of aging.

It remains unclear why only certain DNA repair mutants show phenotypes related to premature aging. It is interesting to note that the DNA repair-deficient mouse models that exhibit reduced health and/or life span in addition to early appearance of age-related phenotypes also display major changes in the expression of liver genes involved in stress response, cell proliferation and apoptosis, glucose and/or lipid metabolism, and inflammatory response. This suggests that NEIL1 (associated with BER), CSB, ERCC1, XPA, XPD (associated with NER), DNA-PKcs/Ku complex (associated with NHEJ), and WRN (associated with NHEJ, HR, or BER) are also implicated (directly or indirectly) with the transcription of a subset of genes (or pathways) important for the aging phenotypes at least in the liver. Such data imply the possibility of targeting specific biochemical pathways (in addition to ROS levels, telomere structural changes, mitochondrial dysfunction) to control or slow down the progression of age-related diseases. The impact of calorie restriction, dietary restriction mimetics, or antioxidants is already under scrutiny in different mouse models of aging [129, 130, 137, 138].
